# Publishing ethics in medical education: guidance for authors and reviewers in a changing world

**DOI:** 10.15694/mep.2020.000048.1

**Published:** 2020-03-26

**Authors:** Richard Hays, Ken Masters

**Affiliations:** 1 James Cook University; 2 Medical Education & Informatics Department

**Keywords:** Medical education, publishing ethics

## Abstract

This article was migrated. The article was marked as recommended.

Medical education publishing is growing rapidly, with both increasing demand for publication space and increasing space availability. The increasing speed of publication, variable degrees of manuscript checking and increasing accessibility pose some challenges to compliance with ethical guidelines for academic publication. In this paper we review the literature and the websites of journals that publish medical education content and present a contemporary view on issues that should be considered by authors, reviewers, editors and readers of medical education publications. Based on this analysis, we present guidance on how to meet desired ethical standards when writing particular categories of manuscripts. Relying on self-judgement of the ethical status by authors may no longer be acceptable. The need to meet ethical guidelines in publishing must be balanced with the desire for freedom of speech and avoidance of editorial bias. Our intention is to provoke discussion and learning within the medical education community of practice.

## Introduction

As medical education grows as an academic discipline, the demand for publication space is also increasing. Careers of junior academics with medical education interests need to develop, and there is a lot of activity within the community of practice ( Wenger, 1998): new medical schools, globalisation of curriculum and assessment practices, and international changes to accreditation practices. Attendance and presentations at medical education conferences continues to grow. Medical education publication is growing, but journals are also becoming stricter on what constitutes good medical education research ( Branch and Kern, 2004; Norman, 2014; Badyal, 2018; Gottlieb *et al.*, 2018). Several more academic publishing outlets have developed to cater for this increased activity, including *MedEdPublish.* While this growth in medical education as an academic discipline is welcome, it is happening as the publishing industry continues to evolve in response to influence from three apparently unstoppable pressures for change.

The first is that the number of communication outlets is increasing substantially, as a combination of technological development and environmental concerns drive a shift to on-line publishing. Authors have more choices about where to submit their work, although prefer more established journals that are recognised by academic institutions and have impact factors. The second change is the explosion in the collection of personal information by most organisations with internet access. Social media sites collect and connect personal data, including family and friendship networks, in ways that many users may not fully comprehend. Most people now have data profiles in several internet locations. While apparently secure and available only to closed user groups, these data are often used for commercial purposes, including marketing, and are often targeted for criminal data hacking purposes. Inappropriate identification of individuals by linking data sources, whether by accident or criminal activity, is a substantial risk, because of the potential for fraudulent activity and defamation action. To mitigate this risk, organisations that gather users’ personal data are now required to meet stricter privacy requirements, such as the General Data Protection Regulation (GDPR) in the EU ( European Commission, 2019).

The third change is the speeding up of publishing processes. Academic medical publishing has existed in some form for over 2000 years. Early collections of symptoms and signs, possible diagnoses, and herbal and surgical treatments contributed to the development and spread of medical knowledge, although circulation was severely constrained because only a few, hand-written copies were available. The invention of printing presses in the 15 ^th^ century allowed for more widespread dissemination and sharing of knowledge, and we now live in a world were there may be only seconds between finishing a document and making it accessible digitally anywhere that there is an Internet connection.

A major challenge to academic publishers is that the increasing number and variety of sources may provide readers with several different, sometimes conflicting, sources of information on the same topic. Whereas publications were once regarded as authoritative for decades or even centuries, based on empirical work of experienced physicians, many publications now have little or no oversight from peers or experts. Self-publication is more common. There are ‘fake’ or ‘predatory’ journals (Grudniewicz A *et al.*, 2019), bogus websites, and reports based on ‘fake’ data. Published information is not necessarily even checked or verified, so much of what we can read may be incorrect, opinion-based, and likely to date rapidly. Hence, while more information is available, readers must determine for themselves what is trustworthy and meaningful.

The journals that we have become used to in academic medicine have developed robust processes for dealing with this situation, with several steps introduced to protect ethical standards. Publishing in medical education has much in common with other forms of academic publishing, but some differences are emerging. This article reports an investigation that arose from the experience of establishing a new, open-peer review academic journal in medical education ( Hays, 2016), aiming to inform authors and reviewers of medical education manuscripts. The resulting information is likely to be relevant to other medical education journals.

## Methods

Three levels of searching were conducted in November and December 2019. First, the author information pages of websites for the six best known medical education journals, in addition to *MedEdPublish*, were searched to identify policies on publishing ethics. Journals included were: *Medical Education,* *Medical Teacher,* *Academic Medicine,* *Teaching and Learning in Medicine,* *Clinical Teacher* and *Education for General Practice.* These journals are relatively new and have strong connections to the large publishing houses, one of which publishes half of them.

Similar information was also accessed for a selection of general medical journals that publish medical education manuscripts from time to time, including: *BMJ,* *Lancet,* *New England Journal of Medicine,* *Journal of the American Medical Association,* and *Medical Journal of Australia.* These journals are older and well-established and tend to be published by the societies that own them.

The websites of all included journals reference the Declaration of Helsinki, Committee on Publication Ethics (COPE) and the International Committee of Medical Journal Editors (ICMJE), so the websites of those organisations were searched for policy documents on publishing ethics.

Searches were also made using Google Scholar and PubMed Central, using the key phrases: “publishing ethics”, “research ethics principles” and “publishing errors”. A predominantly descriptive analysis was conducted by the authors, producing an overview of available publishing ethics policies and practices in medical education publishing, and then distilling these into contemporary advice for authors, reviewers and readers.

## Results: the scope of academic publishing ethics

The inspection of the 12 named journals found that only nine different publishers were involved, due to recent mergers of publishing houses. Policies appeared to be identical across all journals in each publisher’s stable and varied little across publishers. All of these publishers stated compliance with known international policies, such as the Declaration of Helsinki, and COPE and ICMJE policies.

Ethical issues are encountered across most aspects of publishing, from commencement of the academic activity through to publication. They begin with ensuring that the academic activity that will be reported meets ethical standards. For research manuscripts, or perhaps any manuscripts that report data collection and analysis, the methods should align with the research questions or hypotheses. Some observers take the view that using inappropriate methods automatically means unethical research, because this wastes resources and inconveniences participants without adding value. There is uniform international agreement that research involving experiments on animals and humans must have appropriate approval from a relevant Ethics Committee or Institutional Review Board (IRB). Usually, the context is a trial of an intervention that may benefit, but also may harm, participants. These issues are covered well in the Declaration of Helsinki ( WMA, 2013). Medical education research rarely involves scientific experiments covered by these declarations, so the OECD policy on social science research may be more relevant ( OECD, 2016). Ethical data bank management issues are covered in the Declaration of Taipei ( WMA, 2016). The Health Research Council in the UK has an on-line tool for determining if a project requires ethics approval, but this is designed for patient-based projects and may not be as reliable for medical education projects (see http://www.hra-decisiontools.org.uk/research/). All of the 12 journals included in our search stated clearly a requirement for formal ethics approval of manuscripts that report formal research. Increasingly, the practice of authors making decisions on the ethical status of their own projects is being questioned, so it may be best to seek advice when reporting all kinds of projects.

Writing the manuscript requires authors to be identified and listed in a logical order (based on contributions or alphabetical) with the contribution of each described. All authors should agree to being identified. This information confirms that the authors are real people who jointly produced and take responsibility for a product. Some guidance is available for what might constitute author contributions and intellectual policy ( NHMRC, 2018, 2019; CASRAI, 2019). There have been examples of the names of better-known authors being added without permission, in the apparent hope that this would increase chances of acceptance, and even of ‘fake’ authors. The relative positions and contributions of more senior and more junior authors may be important. For example, the practice of senior people automatically being named as an author of everything published by their group, even when they contributed little or nothing, is open to question. An acknowledgement is sometimes more appropriate. Potential conflicts of interest (sometimes described as ‘competing interests’) are relatively common in the relatively small medical education community, and it is better to list all that may apply so that readers can make judgements.

Editorial and pre-publication peer review are useful gatekeeping processes to ensure a high standard of academic work and are also safeguards against unethical publishing. They are, however, fallible. There are numerous examples of high-quality journals publishing questionable articles (Andrew Wakefield’s notorious paper ( Wakefield *et al.*, 1998) was published in the *Lancet,* and took 12 years to be retracted ( The Editors of The Lancet, 2010), and the website *Retraction Watch* ( https://retractionwatch.com/) is filled with papers retracted on an almost weekly basis, frequently because of ethical mis-conduct).

Conversely, editorial and reviewer bias, lack of knowledge, insight and other factors may cause ground-breaking articles to be delayed or even rejected: Peter Ratcliffe’s work, for which he was eventually awarded the Nobel Prize in Physiology or Medicine ( Nobel Foundation, 2019), was originally rejected by *Nature.* This is not a unique incident, as similar incidents had happened to other luminaries, such as Enrico Fermi, Murray Gell-Mann and Hans Krebs ( Italie, 2019). Further, a small proportion of reviews are ‘unprofessional’, in that they may cause offence without providing sound feedback ( Silbiger and Stubler, 2019). A major reason for the establishment of *MedEdPublish* was to introduce open, post-publication peer-review to both capture the value of peer review and reduce editorial bias in medical education publishing. However, this introduces the challenge of maintaining high ethical standards in the reviews and responses, as the communication threads are visible to all readers; this explains the ‘report abuse’ function for each review in MedEdPublish.

## Challenging issues in publishing ethics

While the policies and processes are relatively clear for papers that report projects that fit with the usual medical research paradigm, some manuscripts and authoring practices in medical education publishing may not fit well.

*Reporting of an evaluation of data from learning activities.* A potential ethical issue arises if the researchers are the teachers and the participants providing data are their learners, because there is a power imbalance that may limit the ability of learners to decline participation, a little like the doctor-patient dynamic. Naming institutions and describing learners risks breaching confidentiality, particularly if numbers are small. These kinds of projects are often regarded as ‘minimal’ or negligible’ risk by ethics committees, so long as the objective is quality improvement and only ‘minor discomfort’ is likely for participants. However, while the evaluation might not need ethics approval, the public dissemination of results may not be exempt. An example of definitions and explanations can be found in policies of James Cook University ( James Cook University, 2020), which still require researchers to seek advice on the matter. Authors should check the policies of their own institution, as the wording may vary.

*Reports from workshops and seminars.* It is not uncommon for authors to plan a conference workshop with the potential afterwards to write a report for publication that summarises the key issues or provides a ‘state of the art’ overview. These reports may be very interesting and useful, but could be construed as representing a form of research, where the ultimate intellectual property may be owned (in part) by all who contributed, even though there may be no prior ethics approval and participants attend on a voluntary basis. This seems to be a very grey area, but authors should avoid the potential for participants complaining about being excluded.

*Understanding plagiarism and intellectual property theft.* This is a complex issue. While it would be expected that authors would rely on previously published work to build a case for their manuscript, care must be taken in ‘how much’ and ‘how’. Similarity-detection software is an early step in the publishing process. The percentage word match is not necessarily the critical issue, as relatively small sections can be the central part of the article. As the software searches most of the internet, similarity with a very wide range of publications, including university assignments and theses, books, conference papers and presentations, newsletters and blog sites, as well as other journal articles.

*Understanding self-plagiarism.* Many authors may feel that it is both appropriate and ‘safe’ to re-use wording from their own previous publications, but it may not be. Authors may not realise that they assigned the copyright of previous papers to the publisher and that their work may not be re-published without the written permission of the original publisher. Some journals offer ‘non-exclusive’ copyright assignment or allow use of a proportion (often 10%) of words in other products, so it is best to check the precise wording of all agreements. Even where copyright remains with the author, merely copying and pasting one’s own work from previous publications without correct citation constitutes self-plagiarism.

*Understanding “Salami slicing”.* This is where authors aim for the “minimal publishable unit”, using the same methods and data in more than one paper, with a different introduction and take on the analysis and interpretation ( Neill, 2008; Norman and Griffith, 2008; Karlsson and Beaufils, 2013). This may be regarded in some institutions as a form of research misconduct.

*Use of live hypertext links to other websites within the text of manuscripts.* This practice may be increasing as more information becomes accessible on-line.Links to permanent DOI resources seems to be acceptable, perhaps even desirable, particularly for references, as readers can be taken directly to those resources, but websites can be interactive, altered, not maintained or even removed after publication. As a result, information can be lost in dead links or readers can be connected to unspecified or unknown websites that may use their data or leave cookies.

*Managing manuscripts that are written poorly.* These manuscripts may be difficult to read, perhaps open to misinterpretation, and may leave the authors open to criticism. A variation is where manuscripts are written by authors in a second, third (or more) language. Is this fair to authors and readers? The cost of copy editing and language editing can be substantial and, to keep publishing costs lower, is increasingly regarded as the authors’ responsibility.

*Managing manuscripts from authors based outside the country of publication.* This is becoming very common as English language publications are more accessible and more readily cited. There may be international variations in legal and ethical requirements for consent, information management and intellectual property, but publishers must obey the rules in the country in which they are based. Even so, it is possible to transgress rules in other countries where the journal is accessed, making the legal situation around complaints quite unclear. Therefore, it is likely that editorial decisions may follow relatively conservative advice.

For most issues there are few absolute answers, but rather uncertain areas where editors must make a judgement on acceptability, potentially leading to some variation across journals. Herein lies the art of academic publishing.

## Ethics guidance for specific kinds of medical education manuscripts

The ethical considerations in publishing medical education manuscripts may differ depending on the type of manuscript, while some strategies apply to all types. A detailed discussion and a useful check-list of ethical issues can be found in AMEE’s new *Guide to ethics in medical education digital scholarship* ( Masters, 2019), but this summary outlines some of the most important concerns.

### Authorship and general manuscript preparation

Authorship (names, order, contributions) should be clear. All authors should follow the principles espoused in the Declaration of Helsinki - avoiding identification of research subjects, obtaining informed consent, doing no harm etc. All authors should support the method(s) used to match the research question or purpose and be happy that the manuscript is written well in the grammatically correct, plain language of the publication. Authors should ask colleagues to provide feedback prior to submission. For authors writing in a second or third language, using professional translators may be wise as poor translation can impair meaning.

Authors should be careful in selecting the type of manuscript during the submission process as this may determine the ethics approval requirements. Research papers will be subject to greater ethics scrutiny, as will all papers that report collection and gathering of data from learners or patients. Manuscripts that report innovations or new education methods may use data, and so be either research or at least service evaluations.

### Manuscripts that report any involvement of animal products in education activities

An example is an evaluation of a workshop where participants suture or inject the skin of animals. Most institutions would require that such learning activities have ethics approval to ensure that the animal products are sourced humanely. If so, the authors of the evaluation paper should state this clearly, ideally citing an ethics approval number.

### Manuscripts that report any involvement of learners as subjects of procedures in education activities

Sometimes students practice physical examination skills (including intimate examinations) on each other, paint or draw surface anatomy on each other, or even inject each other with normal saline or local anaesthetic. Increasingly, such activities are being replaced with either volunteer or paid simulated patients, where employment agreements include relevant permissions. A further example is recording of learners’ conducting patient consultations. Most institutions would require that such learning activities have ethics approval. If so, the authors of the evaluation manuscript should state this clearly, ideally citing an ethics approval number.

### Manuscripts that report any involvement of patients

This may be relevant where learners are exposed to patients for learning, such as using real patients in a clinical examination, where there is a risk of causing pain or tiredness. For example, how many times can a soft, enlarged liver be prodded safely? This kind of activity is common, perhaps unavoidable, in academic medical centres. Permission may be implied by being in a teaching health facility, but only explicit permission is likely to be defensible if problems arise. Again, ethics approval should be obtained and stated, ideally with an ethics approval number.

### Manuscripts that report ‘service evaluations’, where learners’ data are used to evaluate educational activities primarily for quality improvement

Steps should be taken to prevent learners being identified; this might be difficult when institution, program, academic year and subject details are provided. Where present, institutional policy documents about any exemption from ethics approval should be cited. Authors should be confident that publishing of reports is included in any ethical approval exemption. The ethics office should be contacted in writing and respond with a clear, written confirmation of exempt status and/or approval to publish from an exempt evaluation. This clear confirmation should be kept along with the data (there may be institutional rules about the duration) in case proof is required.

### Manuscripts that include images

Images of medical education activities can add interest to manuscripts, but increase the risk of identifying participants or offending readers. Increasingly, journals are accepting video clips as appendices to manuscripts. The use of any image of participants requires explicit consent from each participant. Authors should state that they have proof of this consent and should be able to provide it on request. If there is any doubt, then the participants’ identity should be obscured. Care should be taken not to use images that could be interpreted as violent, discriminatory or potentially offensive to readers. All images sourced externally require evidence of copyright permission.

### Manuscripts that report outcomes of conference workshops or symposia

It could be argued that the results of an interactive discussion in an online forum (including blogs and pre-print servers) or at face-to-face conferences and symposia is a form of research, and that the results are the joint intellectual property of all participants. Authors should advise participants about the intention to write a manuscript based on the discussion and obtain written consent from each participant. It may be possible for all participants to be acknowledged by name in the manuscript, should they request this, and even for some participants to be co-authors if they make a substantial contribution to the process.

### Manuscripts that report reviews based on other primary information sources

There are many kinds of reviews, involving qualitative or quantitative methods. Ideally, reviews are a robust form of research, with clear, named search terms, information sources and inclusion criteria, followed by a robust analysis of information in articles that make the final selection. At the other end of the spectrum, there are manuscripts that are largely opinion-based, with references that support the opinion. The former is the stronger model and could be categorized as research manuscripts, although this means that ethics approval may be required. Otherwise, reviews may not need ethics approval although this may depend on the topic. Authors should consult their local Ethics Committee for advice and provide that advice to the journal. This paper, arguably, lies somewhere in the middle of the spectrum.

### Manuscripts that are ‘Case Reports’

Potentially interesting manuscripts can be based on descriptions of an incident, or a series of incidents, in an education context, with some commentary about lessons learned. Possible topics include innovations in teaching and learning, reactions of learners, patients or teachers, or even examples of professional behaviours. Care must be taken to prevent inadvertent identification of individual participants (see above) and institutional permission is advisable if there is any risk of impact on reputation.

### Manuscripts that are editorials, commentaries, letters or personal opinion

These should use no primary data and so usually do not require formal ethics approval. An exception is where authors are requested to revise a research paper into a research letter, as a more concise format. Authors should be prepared to explain further or defend their opinions if required, so should keep supporting evidence. These manuscripts may be provocative, but not insulting, discriminatory or showing bias. While the countries where publishers are based may have freedom of speech protection, published articles can be accessed by readers in places with weaker protection.

## Summary

While the central principles of publishing ethics may have changed little, their interpretation and application are being challenged by the increasing diversity of publishing formats, the ubiquitous ability to publish content rapidly and with limited oversight, and data protection requirements. In this era of information explosion, publishers of academic medical education articles share with our community of practice a responsibility to observe ethical publishing guidelines, to be open and clear about authorship and contributions, to prevent participant identification and to avoid causing unnecessary harm or offence to participants or readers. Ethical publishing has traditionally relied on authors’ making correct judgements about compliance with ethical guidelines, but this may no longer be sufficient for many categories of manuscripts. If there is any doubt, a formal opinion from the appropriate ethics committee should be sought. Authors need to be aware that their declarations of ethical compliance are public statements with some legal standing. On the other hand, freedom of speech remains an important attribute of academic debate and care must be taken to avoid introducing another form of publication bias - inappropriate fear of making an error (IFOME). Getting the balance right can be challenging and ultimately relies on the judgements of experienced researchers, authors, reviewers and editors.

## Take Home Messages

• Ethical issues to consider in medical education publishing are similar to those in other fields of academic medicine, but there are some important differences.
• Ethical publication requires attention to all stages of project design, implementation and reporting.
• It may no longer be appropriate for authors to be the sole judges of the ethical status of their academic work.

## Notes On Contributors

Richard Hays MBBS MD PhD is an experienced medical educator at James Cook University, Australia, who combines clinical practice with academic interests in curriculum and assessment design, implementation and evaluation. For transparency, he is the Editor-in-Chief of MedEdPublish. ORCiD: https://orcid.org/0000-0002-3875-3134

Ken Masters is Associate Professor in Medical Informatics at Sultan Qaboos University, Oman. For transparency, he is Associate Editor of MedEdPublish. ORCiD: https://orcid.org/0000-0003-3425-5020

## Declarations

The authors are Editors of MedEdPublish.
