# Supplementary material for: Publishing ethics in medical education: guidance for authors and reviewers in a changing world
Source: MedEdPublish (2016). 2020 Mar 26;9:48. [Version 1] doi: 10.15694/mep.2020.000048.1 (PMC10697547; doi:10.15694/mep.2020.000048.1)
Supplement: Peer review history

# Reviewer report: Reviewer response for version 1

**DOI:** 10.21956/mep.19995.r30450

**Published:** 2020-05-14

**Authors:** Ngusha Anyam

**Affiliations:** 1 University of Ibadan

This review has been migrated. The reviewer awarded 5 stars out of 5 Profound article that provides a practical, excellent and unbiased view on publishing ethics. I would recommend this paper to all editorial panel members for it's appropriate guidelines.

Reviewer Expertise:

NA

No decision status is available

# Reviewer report: Reviewer response for version 1

**DOI:** 10.21956/mep.19995.r30449

**Published:** 2020-04-03

**Authors:** Shaoting Feng

**Affiliations:** 1 The First Affiliated Hospital of Sun Yat-sen University

This review has been migrated. The reviewer awarded 5 stars out of 5 What an excellent paper! It's so important and practical to us! I would like to recommend this great paper to my colleagues who are interested in medical education.

Reviewer Expertise:

NA

No decision status is available

# Reviewer report: Reviewer response for version 1

**DOI:** 10.21956/mep.19995.r30451

**Published:** 2020-04-01

**Authors:** Tharin Phenwan

**Affiliations:** 1 University of Dundee

This review has been migrated. The reviewer awarded 5 stars out of 5 This is a great article that provides a framework that we may have overlooked when conducting a reseach. It is comprehensive, very practical, and can be applied to other fields outside medical education too. I highly recommend everyone who is interested to conduct research to read it.

Reviewer Expertise:

NA

No decision status is available

# Reviewer report: Reviewer response for version 1

**DOI:** 10.21956/mep.19995.r30447

**Published:** 2020-03-27

**Authors:** Katharine Boursicot

**Affiliations:** 1 Duke-NUS Medical School

This review has been migrated. The reviewer awarded 5 stars out of 5 Excellent overview paper on an important topic. Good to have clear guidelines to disseminate to colleagues undertaking scholarship activities in medical education.

Reviewer Expertise:

NA

No decision status is available

# Reviewer report: Reviewer response for version 1

**DOI:** 10.21956/mep.19995.r30454

**Published:** 2020-03-26

**Authors:** David Taylor

**Affiliations:** 1 Gulf Medical University, Ajman, UAE

This review has been migrated. The reviewer awarded 5 stars out of 5 This is a really important paper, which collates a lot of information which all authors (old and new) need to master. The key point is that everything we do has ethical consequences, and we need to be mindful of them, from inception of a study to final publication. Like others, I will be expecting my graduate students to read, mark, learn and inwardly digest this!

Reviewer Expertise:

NA

No decision status is available

# Reviewer report: Reviewer response for version 1

**DOI:** 10.21956/mep.19995.r30453

**Published:** 2020-03-26

**Authors:** P Ravi Shankar

**Affiliations:** 1 American International Medical University

This review has been migrated. The reviewer awarded 5 stars out of 5 I read with great interest the article by the Editors of MedEdPublish. I have been associated with the journal as a reviewer and a contributor for some time. MedEdPublish provides in many ways an ‘unconventional’ approach to publishing in medical education and the open access policy of the journal increases access. The journal also publishes a broader range of work compared to more conventional journals. The authors have addressed a number of issues with regard to publishing medical education research. The issue of the unequal power balance between teachers and students is an important one. Most often due to a number of reasons teachers judge the effects of their own innovations or teaching programmes. This is not as uncommon as we think. In clinical trials most often we are asking pharmaceutical companies to show that their product is better than a placebo or the standard treatment. The implications of this has been widely written about in the literature. Initiatives have been undertaken to increase the objectivity of clinical trials. In most institutions finding an independent evaluator for an intervention is difficult. The other major issue which the authors have pointed out is the ‘fear’ among students of being identified. This is especially true in small student populations and where demographic information is also collected during the study, increasing the possibility of respondents being identified. I also found the issue of writing reports about conferences and workshops to be of interest. The Clinical Teacher often uses images of teaching sessions and activities in the journal and have a clear policy regarding permissions to be obtained while using images. English is often not the first language of many authors and authors from the developing world may find it difficult to avail the services of paid copy editors. The internet has increased the number of publications options and blog posts, preprints and even Facebook posts are increasingly being used to share information. This is an important article which will be of interest to all medical educators.

Reviewer Expertise:

NA

No decision status is available

# Reviewer report: Reviewer response for version 1

**DOI:** 10.21956/mep.19995.r30452

**Published:** 2020-03-26

**Authors:** Trudie Roberts

**Affiliations:** 1 Leeds Institute of Medical Education

This review has been migrated. The reviewer awarded 5 stars out of 5 An excellent paper which is both interesting and extremely practical in its content. I will certainly be recommending this to both my UG and PG research students. Thank you

Reviewer Expertise:

NA

No decision status is available

# Reviewer report: Reviewer response for version 1

**DOI:** 10.21956/mep.19995.r30448

**Published:** 2020-03-26

**Authors:** Subha Ramani

**Affiliations:** 1 Harvard Medical School, Brigham and Women's Hospital

This review has been migrated. The reviewer awarded 5 stars out of 5 This is an excellent article that I read with interest for myself as an educational researcher and scholar. I strongly recommend that all educators and educationalists, who intend to write, review these guidelines.Regarding ethical approval- I have heard educators who assume their research is exempt from ethical review. The decision to exempt needs to come from ethical boards. I learned this from senior researchers and scholars and it is a good lesson.Quality improvement- I have also heard people stating that their educational project is QI and does not need ethical approval. Based on advice from experts, I now pass on the message that it may be QI for local reporting, but it is scholarship when intent to disseminate steps in (presenting or publishing).Images- There are now several online companies where you can subscribe or pay to get license for specific images. Once license is obtained, we can use these images. I myself use shutterstock, though there are similar companies. One could also use a graphic illustrator, they are not always expensive and could be most useful.International community of scholars- Ethical review procedures are different in different countries and publishers need to pay attention and perhaps require some uniformity in ethical approval. I agree that methodology and ethics are different and need to be judged individually.I learned a lot reading this article and strongly recommend that everyone who intends to publish any educational articles should review this carefully. Some self-policing is needed.Finally, more collaborations and mentoring from seasoned scholars and leaders can ensure that novice scholars publish and publish ethically.

Reviewer Expertise:

NA

No decision status is available

# Reviewer report: Reviewer response for version 1

**DOI:** 10.21956/mep.19995.r30446

**Published:** 2020-03-26

**Authors:** Trevor Gibbs

**Affiliations:** 1 AMEE

This review has been migrated. The reviewer awarded 5 stars out of 5 As publishing ethics become more important this paper is published at a very appropriate time. However, as the subject becomes more important it seems that it becomes more complicated and frequently obtuse; another reason why this paper is of value. As an Associate Editor of several journals, my feeling is that many authors try to get away without considering any ethical considerations in their paper, and are frequently successful, hence my hope that this paper is read by all editorial panel members . The authors have put a lot of work into this paper and their observations and actions are easy to follow and I would recommend this paper to all involved in academic writing and publishing.

Reviewer Expertise:

NA

No decision status is available
